# A Review of Plant-Based Drinks Addressing Nutrients, Flavor, and Processing Technologies

**DOI:** 10.3390/foods12213952

**Published:** 2023-10-29

**Authors:** Aijun Xie, Yushi Dong, Zifei Liu, Zhiwei Li, Junhua Shao, Mohan Li, Xiqing Yue

**Affiliations:** 1 College of Food Science, Shenyang Agricultural University, Shenyang 110866, China; shaojh024@163.com; 2 Department of Chemical and Biomolecular Engineering, National University of Singapore, Singapore 119077, Singapore; axie26@wisc.edu; 3 Department of Food Science and Technology, National University of Singapore, Singapore 117542, Singapore; e0506474@u.nus.edu; 4 Department of Nutritional Sciences, King’s College London, London SE19NH, UK; k21070993@kcl.ac.uk; 5 Jiangsu Key Laboratory of Oil & Gas Storage and Transportation Technology, Changzhou University, Changzhou 213164, China; lzw@cczu.edu.cn

**Keywords:** plant-based drink, flavor, nutrients, processing technology

## Abstract

Plant-based drinks have garnered significant attention as viable substitutes for traditional dairy milk, providing options for individuals who are lactose intolerant or allergic to dairy proteins, and those who adhere to vegan or vegetarian diets. In recent years, demand for plant-based drinks has expanded rapidly. Each variety has unique characteristics in terms of flavor, texture, and nutritional composition, offering consumers a diverse range of choices tailored to meet individual preferences and dietary needs. In this review, we aimed to provide a comprehensive overview of the various types of plant-based drinks and explore potential considerations including their nutritional compositions, health benefits, and processing technologies, as well as the challenges facing the plant-based drink processing industry. We delve into scientific evidence supporting the consumption of plant-based drinks, discuss their potential roles in meeting dietary requirements, and address current limitations and concerns regarding their use. We hope to illuminate the growing significance of plant-based drinks as sustainable and nutritious alternatives to dairy milk, and assist individuals in making informed choices regarding their dietary habits, expanding potential applications for plant-based drinks, and providing necessary theoretical and technical support for the development of a plant-based drink processing industry.

## 1. Introduction

The demand for plant-based alternatives to dairy milk has surged tremendously in recent years, driven by factors including ethical concerns, environmental sustainability, and health-conscious lifestyles [1]. Consequently, plant-based drinks have emerged as a diversity of popular choices for individuals seeking a dairy-free alternative that aligns with their values and offers a range of nutritional benefits [2].

Plant-based drinks, also known as non-dairy or alternative drinks, refer to beverages derived from plant sources, including nuts, grains, legumes, and seeds [3]. These milk alternatives have gained significant attention as viable substitutes for traditional dairy milk, providing options for those who are lactose intolerant or allergic to dairy proteins, and those who adhere to a vegan or vegetarian diet [4,5,6]. In recent years, as the market for plant-based drinks has rapidly expanded, a wide variety of options, including almond, soy, oat, rice, and coconut drinks, have become available to meet the demand. Each variety possesses unique flavor, texture, and nutritional composition characteristics, offering consumers a diverse array of choices to suit their individual preferences and dietary needs [7].

One of the key factors driving plant-based drink popularity is its perceived sustainability [8]. The environmental impact of dairy milk production, including land and water use, greenhouse gas emissions, and animal welfare concerns, has prompted consumers to seek alternative options [9]. Plant-based drink production generally requires fewer resources and generates fewer emissions than dairy milk production, making it a more environmentally friendly option [10]. Plant-derived drinks offer numerous nutritional benefits. While dairy milk is commonly associated with calcium and protein content [11,12,13], plant-based alternatives also provide essential nutrients including vitamins, minerals, healthy fats, and fiber [2]. The nutritional profile varies across different plant-based drink varieties, and fortified options are available to enhance the nutrient content and mimic the benefits of dairy milk [14]. The choice between plant-based drinks and dairy milk for infants and adults depends on individual dietary preferences and nutritional needs, with plant-based drinks offering lactose-free and vegan-friendly options, while dairy milk provides a nutrient-rich source of essential nutrients like calcium, vitamin D, and high-quality protein. Caregivers should prioritize breast milk or infant formula for infants, and adults should select the option that aligns with their dietary and health goals, considering factors such as lactose tolerance and environmental impact.

In this review, we aimed to provide a comprehensive overview of plant-based drinks, exploring the available types and their nutritional compositions, health benefits, potential considerations when choosing a product, processing technologies, and challenges facing the plant-based drink processing industry. We delve into the scientific evidence supporting the consumption of plant-based drinks, discuss their potential roles in meeting dietary requirements, and address limitations and concerns associated with their use. In doing so, we hope to expand awareness of the growing significance of plant-based drinks as sustainable and nutritious alternatives to dairy milk, thereby assisting individuals in making informed choices regarding their dietary habits, expanding potential applications for plant-based drinks, and providing necessary theoretical and technical support for the development of a plant-based drink processing industry.

## 2. Classification of Plant-Based Drinks

Plant-based drinks can be classified according to the raw materials used, such as almonds, oats, soy, walnuts, peanuts, coconuts, cashews, rice, hemp, and flax (Table 1). Plant-based drinks can be broadly classified into six types based on raw materials: cereals (oats and rice), legumes (soybeans and peas), pseudo-cereals (quinoa), seeds (peanuts, sesame, and sunflower), nuts (walnuts and almonds), and high-protein or fatty fruits (coconut). Beverages made from plant-derived ingredients and plant proteins are also referred to as plant-based drinks [7]. Plant-based drinks provide supplemental proteins, calcium, and various other nutrients. They can be consumed in pure form or used as a companion for coffee and tea, and can serve as an ingredient in processed foods (baked products and plant-based ice cream).

## 3. Plant-Based Drink Nutrients

### 3.1. Protein

In the absence of added exogenous protein, soy-based plant drink has the highest protein content, similar to that of cow milk. Soy-based plant drinks are generally considered to be a complete protein source for adults, containing all essential amino acids. Other plant-based drinks have lower protein contents, with rice-based plant drinks having the lowest protein content [16,17]. Compared to milk protein, plant-based drinks may exhibit slight quantitative deficiencies in some essential amino acids, such as methionine and lysine [18]. Methionine and cysteine are the limiting amino acids in pea and soy tissue proteins, whereas lysine is the limiting amino acid in flax proteins and other cereal proteins, including rice [19]. Due to the limited presence of these amino acids, plant proteins are generally considered to provide a lower nutritional value than animal proteins. Additionally, the presence of plant-derived anti-nutritional factors, including phytic acid and saponins, results in the lower digestibility of plant proteins relative to milk protein. Overall, the biological value (BV) and digestible indispensable amino acid scores (DIAASs) of plant proteins are slightly lower than those of milk proteins. For example, BV scores for milk protein and casein are 104 and 80, respectively, whereas BV scores for soy, pea, and flaxseed proteins are 74, 65, and 77.4, respectively. DLAASs for milk protein and casein are 115 and 111, respectively, whereas DLAASs for soy and pea proteins are 89 and 80, respectively [16,19]. Besides protein, other compositions of plant-based drinks can be seen in Table 2.

### 3.2. Dietary Fiber

Dietary fiber refers to the non-digestible carbohydrates present in plant-based foods [27]. It primarily exists in plant cell walls and includes cellulose, hemicellulose, and soluble fibers. Dietary fibers remain structurally intact as they pass through the digestive tract because they are not broken down by digestive enzymes in the human body [28]. Dietary fibers offer various health benefits, including promoting gastrointestinal health, preventing constipation, controlling blood sugar and cholesterol levels, providing a feeling of fullness, and regulating body weight. Common sources of dietary fiber include whole grains, vegetables, fruits, legumes, nuts, and seeds. It is recommended that adults consume 25–30 g of dietary fiber per day to maintain good health [29,30,31,32]. In plant-based drinks, various soluble or insoluble dietary fibers present in the cell walls of seeds, cereals, or fruit-based ingredients, such as flaxseed gum, almond polysaccharides, and soy polysaccharides, exhibit potential prebiotic characteristics that are beneficial to human health. This prebiotic function is not inherent to dairy milk. Prebiotics are compounds resistant to digestion in the small intestine, and select dietary fibers, such as inulin, oligofructose, and fructooligosaccharides (FOSs), fall into this category. They play a prominent role in regulating the gut microbiota and promoting gut ecosystem health [33,34]. This microbial fermentation process generates short-chain fatty acids (SCFAs), notably butyrate, acetate, and propionate, which confer various health advantages, including trophic effects on colonic epithelial cells and the mitigation of inflammatory responses. Additionally, prebiotic fibers foster gut microbiome diversity by providing diverse nutritional substrates for a spectrum of bacterial strains. A balanced and diverse gut microbiome is associated with improved gastrointestinal function, enhanced immune responses, and potential implications for neurological health. More specifically, beta-glucan in oat drinks increases satiety and lowers blood glucose and cholesterol levels [7]. Flaxseed drink contains flaxseed gum, which improves the gut microbiota, controls weight, enhances satiety, and protects gut and cardiovascular health. Soy drinks containing fiber can lower plasma cholesterol in animals or humans without affecting the absorption of essential mineral elements, including zinc and copper. It also helps maintain gut health and controls blood sugar and lipid levels [35].

### 3.3. Fats and Fat-Soluble Ingredients

Plant-based drinks primarily contain unsaturated fatty acids with generally low levels of saturated fatty acids and no cholesterol. This composition is beneficial for lowering low-density lipoprotein and cholesterol levels, thus providing positive effects to individuals addressing high blood cholesterol and cardiovascular diseases [36]. Polyunsaturated fatty acids (PUFAs), a subgroup of dietary fats encompassing omega-3 and omega-6 fatty acids, confer a spectrum of health benefits when judiciously incorporated into the diet. These benefits are underpinned by intricate biochemical mechanisms. Omega-3 PUFAs, notably found in fatty fish and flaxseeds, substantiate reduced risks of cardiovascular disease through the mitigation of triglyceride levels, blood pressure regulation, and anti-inflammatory actions. Furthermore, omega-3 PUFAs exhibit anti-arrhythmic properties, modulating heart rhythm and averting arrhythmias. In terms of cognitive well-being, these PUFAs, particularly docosahexaenoic acid (DHA), assume a pivotal role in brain development and function, enhancing cognitive faculties and conferring protection against neurodegenerative conditions, such as Alzheimer’s disease. Additionally, mood regulation is influenced by omega-3 PUFAs, which may ameliorate symptoms of depression and anxiety. Their robust anti-inflammatory attributes extend to the management of chronic inflammation, impacting the pathogenesis of prevalent ailments including cardiovascular disorders, arthritis, and certain cancers. Beyond these, omega-3 PUFAs underpin various other health benefits, including skin barrier fortification, immune system augmentation, eye health preservation, potential cancer risk reduction, appetite regulation for weight management, and vital contributions to pregnancy and child development. To optimize these benefits, maintaining an appropriate balance between omega-3 and omega-6 intake is pivotal, as excessive omega-6 and insufficient omega-3 consumption can contribute to inflammatory states and health complications. Consequently, a balanced diet integrating sources of both omega-3 and omega-6 PUFAs is advocated for comprehensive health promotion. Overall, higher proportions of PUFAs, especially n-3 PUFA, other bioactive essential fatty acids, and fat-soluble bioactive components, contribute to the prominent health benefits of plant-based drinks (Table 3).

### 3.4. Vitamins and Minerals

Most plant-based drinks are rich in minerals including calcium, magnesium, selenium, potassium, zinc, phosphorus, copper, and manganese (Table 4). For example, the respective almond and soybean calcium contents are approximately 269 and 277 mg/100 g, whereas the respective magnesium contents are approximately 270 and 280 mg/100 g. The respective potassium contents are approximately 733 and 1797 mg/100 g. The endogenous calcium content of plant-based drinks varies widely among products, but when fortified with external sources of calcium, the calcium content in plant-based drinks is typically higher than that in cow’s milk [43]. In general, the absorption rate of calcium carbonate used for calcium fortification of plant-based drinks is high, although calcium carbonate is prone to precipitation, which reduces calcium bioavailability in plant-based drinks [44,45]. Cow’s milk is considered an excellent source of vitamins; however, it has a low vitamin D content and usually requires external supplementation for fortification (Table 5). Plant-based drinks also tend to have low vitamin D content. Therefore, commercially available plant-based drink products are often fortified with vitamin D. Soy drinks and almond drinks also have an advantage over cow’s milk in vitamin E content, with levels reaching approximately 4.0 and 3.84 mg/100 mL, respectively. Coconut, almond, and cashew drinks have vitamin A contents exceeding 60 μg/100 mL, approximately twice the amount found in cow’s milk [46]. Overall, plant-based drinks have an advantage over cow’s milk in terms of fat-soluble vitamin content, whereas water-soluble vitamins may require fortification.

### 3.5. Bioactive Molecules

Plant-based drinks generally contain beneficial bioactive molecules, including flavonoids, phenolic acids, lignans, and phytosterols (Table 6) [47]. Plant polyphenols exhibit excellent antioxidant properties and provide significant health benefits, including anticancer effects, protection against radiation damage, antimicrobial activity against pathogenic bacteria, lipid-lowering effects, and the prevention of cardiovascular diseases. Resveratrol in peanut drinks reportedly possesses antioxidant, antibacterial, hepatoprotective, cardiovascular, radioprotective, and anti-HIV activities [48]. Sesamol in sesame drinks has been reported to inhibit obesity and insulin resistance in mice fed a high-fat, high-fructose diet. It reduces hepatic fat synthesis, inhibits lipid accumulation and inflammatory responses in white adipose tissue, and decreases adipocyte size while promoting the conversion of white adipose tissue to brown adipose tissue by improving mitochondrial lipid metabolism [49]. Lignans, such as enterolignans, are natural plant estrogens found in high amounts in flaxseed drinks. They can control the production of three types of estrogen, inhibit ovarian estrogen production, reduce the risk of breast cancer, and exhibit significant anti-colorectal cancer effects. Their antioxidant function primarily centers on the neutralization of free radicals, highly reactive molecules capable of inducing cellular damage, including DNA, protein, and lipid harm, potentially culminating in cancer and other chronic maladies. The mechanistic underpinnings of lignan-mediated anti-cancer effects encompass several facets: free radical scavenging via electron donation, the modulation of inflammatory processes, participation in estrogen metabolism pathways (particularly enterolignans), interference with key cellular signaling pathways implicated in tumorigenesis, the induction of apoptosis, and the regulation of angiogenesis. Moreover, lignans may enhance detoxification processes via the upregulation of detoxifying enzymes. Consequently, a growing body of research suggests that lignans may be associated with a diminished risk of specific cancer types, particularly hormone-related malignancies like breast, prostate, and ovarian cancers [50]. The role of flavonoids within plant-based drinks hinges upon their natural occurrence in constituent ingredients or their introduction during processing. Certain plant-based drink varieties, such as almond and soy drinks, may contain inherent flavonoids derived from their foundational ingredients, endowing these beverages with antioxidant capabilities. These flavonoids possess the capacity to mitigate oxidative stress and confer potential health benefits. However, it is important to note that flavonoid enrichment is not a prevailing practice in commercially available fortified plant-based drinks, which predominantly focus on the fortification of vitamins and minerals to replicate the nutritional profile of dairy milk. Therefore, individuals with specific health risks, such as those at a high risk of cardiovascular diseases, may experience enhanced benefits from consuming plant-based drinks.

## 4. Key Substances That Contribute to Aroma in Plant-Based Drink

Flavor is an important quality of plant-based drink that determines its market acceptance [62]. Flavor is determined through small molecules formed via the decomposition of proteins, fats, or carbohydrates in a food. These substances can stimulate the sensory neurons responsible for smell and taste, creating a comprehensive physiological sensation. Molecules contributing to flavor can be divided into odorants and tastants. Tastants are nonvolatile food compounds that are perceived by taste buds in the oral cavity, whereas odorants are volatile food compounds that are sensed by olfactory receptors in the nasal mucosa [63,64,65]. Enhancing aroma and reducing or mitigating the generation of off-flavors are key aspects of flavor modulation in plant-based drinks. Selecting high-quality ingredients and employing environmentally friendly processing techniques to prepare plant-based drinks with excellent flavor significantly impacts market potential. With advancements in flavor extraction and separation technologies, the most widely used techniques for volatile compound extraction include headspace solid-phase microextraction (HS-SPME), dynamic headspace sampling (DHS), solvent-assisted flavor evaporation (SAFE), stir bar sorptive extraction (SBSE), liquid–liquid extraction (LLE), and simultaneous distillation extraction (SDE). These techniques are often combined with gas chromatography–mass spectrometry (GC-MS) and olfactory techniques, such as gas chromatography–olfactometry (GC-O), aroma extract dilution analysis (AEDA), and dynamic headspace dilution analysis (DHDA), for aroma and off-flavor identification in plant-based drinks [66,67,68].

Different volatile compounds exhibit distinct odor characteristics and contribute to the overall flavor profile of a plant-based drink when combined in certain proportions. Based on the existing literature, the main aromatic compounds contributing to the fragrance of plant-based drinks include aldehydes, ketones, alcohols, esters, and acids [67,69].

### 4.1. Aldehyde Compounds

Aldehyde compounds are primarily formed through the degradation and auto-oxidation of unsaturated fatty acids such as oleic and linoleic acids. Aldehydes generally have low odor thresholds and contribute significantly to flavor profiles, making them important aromatic compounds [70]. Medium-chain aldehydes exhibit fatty, fresh, and greasy aromas, whereas aldehydes with higher carbon numbers produce a citrus peel aroma [71]. During soybean growth and processing, over 20 volatile compounds are associated with a beany off-flavor, with hexanal being the most important [72,73]. The hexanal content in walnut kernels correlates negatively with nutty and sweet aromas, and correlates positively with bitterness and acidity. Therefore, storing walnuts at 5 °C in a light-protected environment is considered optimal [74]. Benzaldehyde, a metabolite of phenylalanine, imparts a fruity aroma to plant-based drinks. In soy drinks, 3-methylbutanal contributes to a dark-chocolate flavor, whereas decanal provides sweet, citrus, and floral tastes. In oat acidophilus drinks, nonanal exhibits a honeywax floral aroma, and octanal produces a strong fruity fragrance [75].

### 4.2. Ketone and Alcohol Compounds

Ketones are another product of fat oxidation. Ketones produce less intense aromas in walnut drinks, contributing less to their flavor. Ketones are generally associated with creamy and fruity aromas. For example, 2-methyl-3-hydroxy-4-pyranone is present in both soy drinks and vanilla soy drinks and evokes a caramel aroma [76]. 2,3-butanedione and its degradation product, 3-hydroxy-2-butanone, generate a sweet aroma in prepared soy drinks and a creamy aroma in roasted walnut drinks, possibly due to prolonged exposure to high temperatures [77]. During the lyophilization of peas, significant amounts of 3,5-octadien-2-one and-ionone are produced, possibly through the oxidative and condensation reactions of carotenoids [78]. Alcohol compounds are primarily produced through the oxidative degradation of fatty acids, such as hexanol, which exhibits a floral aroma and is a product of linoleic acid auto-oxidation. 2,3-butanediol contributes to the fruity aroma of walnut drinks, whereas the nonanol present exclusively in walnut drinks roasted for 25 min imparts a fresh fatty note [77]. Nonanol in soy drinks has a strong rose and orange throat aroma, along with a fresh fatty note [79]. Vanillin and linalool are present in vanilla drinks, and have distinct and unique aromatic profiles.

### 4.3. Ester and Acid Compounds

Esters are formed via the esterification of carboxylic acid derivatives with alcohols and are primarily derived from the oxidation of lipid precursors [80]. Esters are characterized by their typical fruity aromas, which contribute to the delicate fragrances of plant-based drinks. The key aromatic ester compounds in roasted walnut drinks are ethyl acetate, triethyl phosphate, and ethyl benzoate, all of which impart fruity notes. Cinnamyl acetate contributes sweet orange and grape aromas, whereas ethyl palmitate imparts a creamy fragrance. The quantity of esters in roasted walnut drinks increases with prolonged roasting time [81]. Acidic compounds contribute significantly to the taste profiles of plant-based drinks. Butyric acid, valeric acid, and l-lactic acid are characteristic acidic flavor compounds in fermented coconut drinks that impart a creamy aroma [82]. In vanilla soy drinks, key acidic compounds include 17-octadecynoic acid, palmitic acid, and oleic acid, whereas in soy drinks, 3-butyne-1-acid, acetic acid, and 15-hydroxydecanoic acid are predominant.

### 4.4. Pyrazines and Other Compounds

Pyrazines are formed primarily via Maillard reactions between reducing sugars and amino acids and via Strecker degradation of carbonyl compounds [83]. Glucose degradation leads to the formation of carbonyl compounds that react with free amino acids to produce α-amino ketones under alkaline conditions. Subsequently, these compounds undergo condensation to form various pyrazine compounds. During plant-based drink preprocessing steps, including microwaving, roasting, and radiofrequency treatments, pyrazines and alkylated pyrazines are generated. These volatile substances can partially mask off-flavors in plant-based drinks [84]. Because of their high concentrations and low odor thresholds, pyrazine compounds exhibit strong roasted, nutty, and caramel flavors. For example, 2,5-dimethylpyrazine imparts a roasted and nutty aroma, whereas 2, 3, 5-trimethylpyrazine imparts a nutty fragrance [77]. Additionally, 2,4-di-tert-butylphenol is a major phenolic aroma compound in soy drinks and is present in significant proportions among the phenolic aroma components. In addition to 2-ethylfuran, soy drinks and vanilla soy drinks also contain other aromatic compounds, including 2-methyl-1-propene, octadecynol, and 8-octadecyne. Phenolic compounds such as 4-ethenylguaiacol have been identified as important aroma contributors in walnut drinks, exhibiting a clove-like aroma. Additionally, 4-allylanisole, which is present in roasted walnut drinks, imparts a fennel-like aroma and is one of the major aromatic components in almond oil [77,85].

## 5. Flavor Formation Pathways

### 5.1. Maillard Reaction

**Figure 1:**
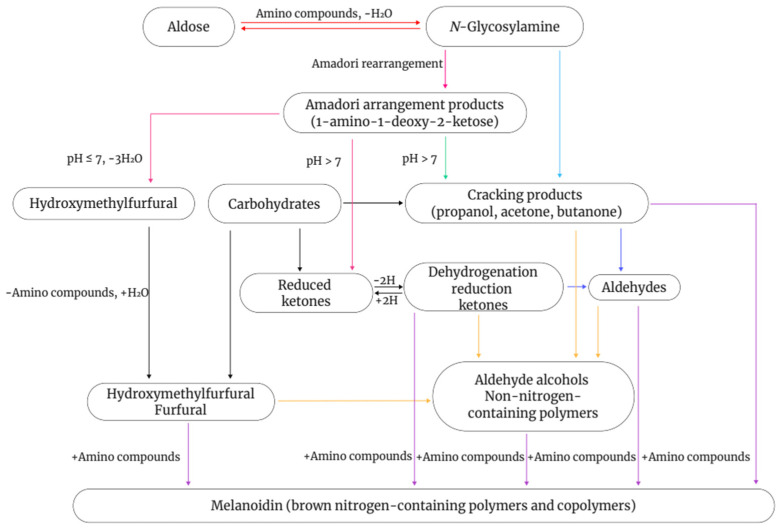
Schematic diagram of the Maillard reaction.

The Maillard reaction is a complex non-enzymatic reaction that involves the condensation of reducing sugars with amino groups in amino acids, peptides, or proteins. It can generate a variety of compounds with different colors and flavors via multiple pathways (Figure 1). This process, also known as non-enzymatic browning, is generally divided into three stages [86,87,88]. In the initial stage, the carbonyl group of the reducing sugar undergoes nucleophilic addition to the amino group of the amino acid to form a Schiff base. Owing to their instability, Schiff bases undergo cyclization to form N-substituted aldimines, which then undergo Amadori rearrangements to form reactive intermediates. To reduce sugars to produce these intermediates, the sugars must be converted into an open-chain structure, which occurs slowly during this stage. Therefore, this stage does not cause significant changes in the color and flavor of food but generates flavor precursors [89]. During the intermediate stage, the Maillard reaction becomes more complex. Intermediates formed in the initial stage, such as Amadori rearrangement products and Heyns compounds, are further degraded to produce reducing ketones, furfural, and unsaturated carbonyl compounds. In the final stage, the numerous reactive intermediates formed in the intermediate stage, including reducing ketones, unsaturated aldimines, and glucosone aldehydes, undergo further condensations, polymerization, or reactions with amino acids, ultimately leading to the formation of melanoidins [90].

As Maillard reaction substrates, amino acids serve as the major determinants of flavor in plant-based drinks. The most significant mechanism of amino acid degradation is Strecker degradation. Different amino acids undergo Strecker degradation to produce specific aldehydes, which are important compounds responsible for distinct flavors in foods, and which serve as intermediates in further reactions. Dimethyl sulfide is formed through the thermal degradation of its precursor, S-methylmethionine, and exhibits a strong boiled cabbage flavor [91]. In addition, during thermal processing of soybeans, their proteins can undergo Maillard reactions with reducing sugars, resulting in food browning and the formation of various volatile compounds. Concurrently, the activity of endogenous oxidative enzymes decreases, inhibiting fatty acid oxidation reactions and reducing the generation of beany-flavor compounds [92,93]. In the absence of lipoxidase, the production of volatile compounds in soy drinks is associated with the Maillard reaction. When lipoxidase activity is lost, major unsaturated fatty acids, including linoleic acid, oleic acid, and linolenic acid, are the primary sources of volatile compounds. This indicates a direct relationship between the Maillard reaction and major unsaturated fatty acids in soy drinks [94].

### 5.2. Oxidative Degradation of Fats

Oxidative fat degradation is the main cause of off-flavors in plant-based drinks. When plant-based drink ingredients are stored under normal conditions, drink quality is relatively stable, but endogenous lipoxidases can be activated during processing, leading to the development of rancidity and other undesirable flavors [95]. Lipid oxidation can be divided into two main pathways: enzymatic and non-enzymatic.

Linear-chain saturated fatty acids undergo oxidation, forming flavor compounds including short-chain or medium-chain fatty acids, aldehydes, alcohols, esters, lactones, and methyl ketones. The oxidation reactions of unsaturated fatty acids, such as linoleic acid and linolenic acid, yield various aldehydes, alcohols, and esters [96,97]. The main off-flavor compounds in soy drinks are C6 and C9 aldehydes and their corresponding alcohols. These compounds are significant constituents of the flavors found in vegetables, fruits, leaves, and leguminous plants, and are primarily derived from the lipoxygenase (LOX) enzyme-catalyzed oxidation pathway [98,99]. LOX oxidizes linoleic acid to form 13- or 9-hydroperoxy-octadecadienoic acid (13-/9-HPOD), which is further cleaved via hydroperoxide lyase to generate hexanal; (E,E)-2,4-decadienal; (E)-2-octenal; and other compounds. When linolenic acid serves as the lipid precursor, (E)-2-hexenal, (E,Z)-3,6-nonadienal, and other compounds are formed [100]. The generated C6 and C9 aldehydes can be further metabolized via alcohol dehydrogenase to produce the corresponding alcohols, such as hexanol and (E)-2-hexenol. Non-enzymatic oxidations, including photooxidation and autoxidation, are primarily caused via heat, light, photosensitizers, oxygen, and transition-metal ions. They can generate volatile compounds including aldehydes and furans, thereby influencing the flavors of plant-based drinks. Light exposure during food processing is unavoidable. For example, after storing soy flour under light for a certain period, its 2-pentylfuran content increases significantly [101]. Singlet oxygen can be generated in the presence of riboflavin in soy drinks, and through specific oxidative mechanisms, riboflavin catalyzes the formation of 2-pentylfuran from linoleic acid. Chlorophyll can induce the formation of singlet oxygen through a similar process, and singlet oxygen content is positively correlated with the duration of exposure to light and air [102].

## 6. Processing Technology

**Figure 2:**
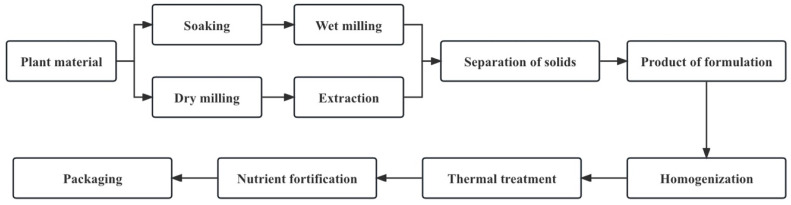
The general workflow of plant-based drink manufacturing processes (adapted from [1]).

A plant-based drink is a colloidal suspension or emulsion consisting of dissolved and decomposed plant material [7]. Although the exact processes vary, the same general outline applies to all plant-based drink processing methods (Figure 2). Water extraction of plant-based drinks can be divided into two types: dry processes (the dry milling of raw materials and the extraction of flour from water) and wet processes (the soaking and wet milling of plant sources) [1].

### 6.1. Pre-Treatment

Before making plant-based drinks, some pretreatment of raw materials may be required, including soaking, blanching, steaming, and baking. The purpose of pretreatment is to enhance extraction, increase nutritional quality, enhance organoleptic characteristics, and eliminate off-odors [6,16].

Soaking is an important pretreatment process in the production of plant-based drinks. The main purpose of soaking is to promote softening and expansion of raw materials including grains and nuts, and to facilitate the breaking of raw materials during the grinding process [103]. This process reduces resistance to mechanical grinding and fully hydrates proteins, rendering them easier to leach and thereby increasing the extraction rate. Soaking also helps reduce the initial microbial load, eliminate off-odors, improve organoleptic properties, and enhance nutritional value [77]. Some raw materials, including cowpeas, cashews, soybeans, oats, and sesame seeds, usually need to be soaked in water for 3–18 h. Some raw materials, including soybeans, peanuts, and tiger nuts, can be soaked in alkaline solutions with 0.2–2% NaHCO3 at ratios ranging from 1:2 to 1:12 to eliminate nutty or beany flavors [7,14,104,105].

Starch in raw materials is gelatinized during steaming or baking, and the peculiar smell of the raw plant materials can be effectively controlled. This process can remove heat-sensitive anti-nutritional factors and toxic and harmful substances (such as cyanogenic glycosides) from raw materials, as well as partially inactivate enzymes. In addition, Maillard and other reactions can occur during baking, which can change plant material flavors and impact aroma development [106,107]. Heating can also reduce protein solubility and extraction rates [108].

### 6.2. Extraction

To improve the yield, extraction efficiency can be increased by increasing the temperature, adding enzymes, fermenting, germinating, or increasing the pH (using NaOH or bicarbonate). During the extraction process, an alkaline medium is used to increase the protein yield. Higher extraction temperatures also increase fat extraction, although protein denaturation reduces fat solubility and yield [1].

Microwaves, such as ultra-high-frequency electromagnetic waves, promote high-frequency reciprocating dipole moments, generating “internal frictional heat”, which is absorbed by food and water to generate heat [109]. It can realize simultaneous heating and temperature increase without fast and uniform heat conduction, and energy consumption is a small fraction of that required in traditional heating [110]. Compared to the steam injection extraction method, microwave-assisted extraction can significantly improve the extraction yield of plant-based drinks, including protein content, total soluble solids, and protein solubility. It completely destroys plant cell and subcellular structure integrity, promoting the release of proteins, lipids, and other soluble solids [111]. Microwave-assisted extraction can also help remove safety risk factors, including cyanogenic glycosides, facilitate aroma enhancement through Maillard reactions, and passivate endogenous oxidases in raw plant materials, to improve the oxidative stability of plant-based drinks [112].

### 6.3. Enzymatic Processing

Enzymolysis technology functions under mild reaction conditions and exhibits low energy consumption, high efficiency, and low solvent consumption, promoting the release of intracellular compounds, increasing soluble sugar and protein contents, and improving product biological activity, thereby improving the stability and sensory qualities of plant-based drinks [113]. Enzymatic hydrolysis is a key step in promoting nutrient dissolution during plant-based drink production. Carbohydrases and proteases are widely used in plant-based food processing [114]. Carbohydrases hydrolyze glycosidic bonds in plant cell wall layers, promoting the breakdown of insoluble fibers, generating low-molecular-weight sugars, and releasing proteins and other intracellular compounds [115]. For example, cellulase hydrolyzes primary cell walls, whereas pectinase hydrolyzes secondary cell walls [116]. The disruption of the network structure of plant cell wall components via pectinase increases the release rate of proteins and fats into cereal beverages. Second, enzymatic hydrolysis can improve the stability and flavor of plant-based drinks. Carbohydrase treatment (1.2% Celluclast 1.5 L, 3 h) improves the physical stability of soybean drinks during storage and ameliorates bean flavor [117]. It is generally believed that proteolysis will release low-molecular-weight peptides, resulting in bitter taste, but when Sahoo et al. immobilized two non-commercial proteases derived from sunflower seeds, added them to soybean drink, and incubated them at 30 °C for 1 h, they found that the beany smell decreased and the pleasant smell increased [118]. Low-molecular-weight peptides produced after proteolysis are primarily composed of hydrophobic amino acids. Hydrophobicity, primary sequence, spatial structure, peptide chain length, and molecular size are all determinants of peptide bitterness. Therefore, specific proteolysis is required to prevent bitterness in plant-based drinks [114]. Enzymatic hydrolysis can also enhance biological activities in plant-based products because this technology can degrade macromolecular substances into smaller molecular compounds with higher biological activity and increase the release of bioactive components (polyphenols, flavonoids, etc.). For example, after protease treatment of soybean drinks and flaxseed protein, hydrolysate showed antioxidant, anti-inflammatory, anti-obesity, and immunomodulatory activities [119]. Additionally, because phenolic compounds in plant materials may form protein–phenolic complexes with globular proteins through hydrophobic interactions and hydrogen bonds, the release of total phenols increases after proteolysis of sesame seed extracts [120].

### 6.4. Fermentation

The production of plant-based drinks using microbial fermentation generally involves mixed-culture fermentation using two or more microbial strains to enhance the fermentation effect and improve the quality of the final product [82]. Lactic acid bacteria, bacilli, and yeasts are the most widely used microorganisms for this purpose [121]. Fermentation can improve plant-based drink flavors by reducing undesirable odors or by creating pleasant aromas. After peanut drink fermentation, the beany flavor was mitigated by the reduction of n-hexanal and n-hexanol, and diacetyl and 2,3-butanedione, with a butterscotch aroma, were produced during fermentation [114]. Fermentation can also improve the nutritional value of plant-based drinks by increasing nutrient content, improving nutrient bioavailability, and removing antinutritional factors [122]. Fermentation can increase protein and vitamin contents [82] as well as improve calcium and vitamin bioavailability. Significant increases in crude protein content and B vitamins, such as riboflavin and thiamine, have been observed in soybean drinks fermented with bifidobacteria [123]. Additionally, vitamins (including vitamins B and K) are produced during the fermentation process. Yeast fermentation promotes vitamin B2 production [82]. In addition, since lactic acid bacteria can produce phytase to catalyze the hydrolysis of phytate into inositol and phosphate, fermentation technology has the potential to reduce the content of anti-nutritional factors in raw plant materials and increase their utilization of minerals [124]. Fermentation also enhances biological activities in plant-based drinks. Fermentation of almond drinks using lactic acid bacteria increases the content of phenolic compounds, conferring higher antioxidant capacity [125]. Soybeans fermented using lactic acid bacteria can produce bioactive peptides that inhibit angiotensin-converting enzymes and display antihypertensive effects [126]; β-glucosidase produced via lactic acid bacteria during fermentation can convert conjugated isoflavones in soy drinks into more bioactive glucoside ketones [127,128].

### 6.5. Germination

Germination is a common method for releasing the nutrients and phytonutrients of plant-based drinks, making them more readily available to digestive enzymes [129]. Sprouted grains are more nutritious than raw grains, and rich in digestible energy, bioavailable vitamins, minerals, amino acids, proteins, and phytochemicals [130]. Germination promotes the enzymatic decomposition of carbohydrates into simple sugars by activating endogenous enzymes such as α-amylase, thereby increasing the digestibility of starch degradation and providing energy for seed development [131]. The effect of sprouting on carbohydrates is largely dependent on the activation of hydrolytic and amylolytic enzymes, resulting in a reduction in starch and an increase in simple sugars [131]. The duration of the process is an important factor. When the amylase activity is at its maximum, the maximum hydrolysis time of starch is 48 to 72 h [132]. Studies have shown that after germination, the phenolic activity and antioxidant activity of millet drink increased by 92 ± 1.99 and 33.42 ± 0.55%, respectively. The amalgamation of sonication with germination reduced the average antinutrient concentration to 23.31 ± 0.36% [133]. The germination process enhances the functional properties of plant-based drinks. The application of germination processes combined with other technologies would be a potential processing technique to acquire nutritious plant-based drinks.

### 6.6. Separation

Separation is a necessary step in plant-based drink production. Its purpose is to separate solid particles and suspended impurities in plant-based drinks. It mainly removes insoluble fibers to ensure that the particles reach a certain fineness, which is beneficial for the stability of the product. According to final product particle size requirements, separation can be achieved via filtration, decantation, or centrifugation to remove coarse particles from the slurry [1]. When using ingredients with a high-fat content, such as peanuts, excess fat can be removed via skimmers during dairy processing. The separated creamy product can be heat-treated to obtain oil or used as an ingredient in ice cream or baking recipes [108].

### 6.7. Product Formulation

The protein, vitamin, and mineral contents in plant-based drinks are very important. Therefore, some plant-based drinks are selectively fortified with exogenous nutrients during processing to increase the total content of specific nutrients. To improve the taste and flavor of plant-based drinks, sugar, salt, acidity adjustment, flavors, and fragrances are also added from exogenous sources during the blending process [3,134]. To maintain a stable plant-based drink system after high-temperature sterilization, emulsion stabilizers, such as monoglyceride fatty acid ester, diglyceride fatty acid ester, sucrose fatty acid ester, carboxymethyl cellulose sodium, and xanthan gum, are used. Although plant-based drinks are similar to animal milk in terms of appearance and consistency, significant differences in nutritional quality and bioavailability exist. Therefore, these products must be fortified to improve their nutritional quality [1]. The nutrients used must be bioavailable and sufficiently stable [108].

### 6.8. Homogenization

Plant-based drinks contain insoluble particles including proteins, starches, fibers, and other cellular materials. These particles are denser than water and, therefore, settle, making the product unstable. Suspension stability can be improved by reducing particle size to increase solubility, or by using hydrocolloids and emulsifiers. Homogenization improves plant-based drink stability by breaking up aggregates and lipid droplets, thereby reducing particle size and increasing their distribution [16]. Mechanical devices used for homogenizing raw materials include high-shear mixers, colloid mills, high-pressure valve homogenizers, and microfluidizers [1].

Ultra-high-pressure homogenization (UHPH) is a common homogenization method used in food processing [135] that is widely used with plant-based drinks. UHPH can significantly improve the physical stability of almond and soy drinks and extend their shelf lives [136]. It offers significant advantages, including the effective inactivation of microorganisms, the retention of micronutrients, green energy saving, and uniform processing [137,138]. In UHPH, the liquid sample is subjected to shear force and cavitation explosion force in the cavity of the homogenizer to produce high-speed fluid impact and vortex action. The structure within the liquid sample is destroyed, and particles within the emulsion become smaller, allowing a more stable suspension, thereby effectively improving the stability of plant-based drinks [139]. Because high pressures can damage microorganisms, UHPH treatment can inhibit the growth of harmful microorganisms and achieve sterilization [126]. Compared with UHT treatment, soybean drinks treated with UHPH (300 MPa, 80 °C) have higher colloidal stability, a stable primary oxidation level, and a significantly lower hexanal value, and can be stored at room temperature for up to 6 months [140]. Both 200 and 300 MPa UHPH reduce the number of spores and Enterobacteriaceae and reduce particle size in plant-based drinks. At 200 MPa, soy drink proteins are partially denatured, whereas at 300 MPa, the degree of denaturation is the same as that observed after UHT treatment [126]. After UHPH treatment, the product has a longer shelf life and better quality characteristics. It is, thus, expected to replace heat treatment in the production of plant-based drinks. UHPH equipment must be combined with sterile filling, requiring strict production equipment monitoring and process control procedures.

### 6.9. Heat Treatment

Heat treatment is applied to kill bacteria and thereby extend shelf life by reducing microorganism numbers and inactivating enzymes [141]. An appropriate combination of time and temperature can ensure the destruction of microorganisms and enzymes in plant-based drinks while avoiding the aggregation of oil-coated droplets at high temperatures [142]. Plant-based drinks usually undergo pasteurization, ultra-high-temperature instantaneous sterilization, high-temperature high-pressure sterilization, or other methods to extend shelf life, but high temperatures can cause changes in the structure and physical and chemical properties of plant-based drinks. In particular, the denaturation of proteins and polysaccharides in a stable solution will destabilize the system, and heating of starch in a system will significantly increase viscosity, which may also negatively impact the quality of plant-based drinks [143,144]. Therefore, the use of new food processing technologies, including pulsed electric fields, to extend shelf life is recommended [108]. The use of emerging food processing techniques, including pulsed electric fields and ohmic heating, has been proposed to prolong the shelf life of plant-based drinks [1].

Pulsed electric field (PEF) is a non-thermal food sterilization technique that exposes samples to pulses of high pressure at temperatures in the range of 30–40 °C [145]. The application of high-intensity PEF (10–80 kV/cm) causes electroporation to increase the permeability of microbial cell membranes, eventually leading to cell damage or death [146]. PEF can inactivate endogenous food enzymes and kill microorganisms, but exerts a less negative impact on food nutrition, texture, taste, and color [147,148]. It is mainly suitable for liquid foods with low conductivity and low viscosity, and has been gradually applied to an increasing number of liquid products, including plant-based drinks, to extend their shelf life [45]. The application of PEF to soy drinks can effectively inactivate Escherichia coli and Staphylococcus aureus without affecting soy drink quality characteristics. This technology may, thus, be a favorable alternative to heat treatment for soy drink pasteurization [149]. PEF treatment and parameter optimization (treatment time, pulse intensity, pulse frequency, and pulse width) also affect enzyme inactivation. Studies have shown that soybean lipoxygenase activity decreases with increasing treatment time, pulse intensity, pulse frequency, and pulse width, and stronger treatment parameters result in a higher degree of soybean lipoxygenase inactivation. The maximum inactivation of soybean lipoxygenase via PEF was 88% at 42 kV/cm (duration, 1036 μs; pulse frequency, 400 Hz; pulse width, 2 μs; temperature, 25 °C) [150]. Additionally, studies have revealed that pulse type and treatment (soaking or cooking) have significant effects (*p* < 0.0001) on soybean trypsin inhibitory activity, and the interactive effect of pulse type-by-treatment was also significant (*p* < 0.0001) [151]. While PEF does not inactivate spores, it can be inactivated by adding organic acids or nisin, adjusting pH, and other methods. Currently, the industrialization of this technology faces certain limitations, including the high cost and limited development of industrial PEF equipment [152].

Ohmic heating is an advanced heat treatment technology that uses a low-frequency electric current to heat food, which can kill spoilage microorganisms and prolong shelf life [153]. When a 50–60 Hz electric current is applied to the food matrix, electrical energy is transported through the resistive medium to promote ion recombination and increase the molecular motion speed to facilitate the release of thermal energy [110]. Ohmic heating can extend the retention of heat-sensitive components, increase production and energy efficiency, and heat quickly and uniformly. Electric field strength, temperature, and time all influence the post-treatment effect [154]. Saxena et al. studied the effect of ohmic heating on polyphenol oxidase activity in sugarcane beverages under three electric field intensities (24, 32, and 48 V/cm) and four temperatures (60–90 °C) with a treatment time of 5–20 min. At 60 °C, polyphenol oxidase activity decreased with increasing electric field strength, while at 70–90 °C, an increase in enzyme activity was observed at 32 and 48 V/cm [155]. This technology has also been used in the production of soybean drinks, where it reduces the beany smell and reduces the activity of endogenous trypsin and chymotrypsin inhibitors due to combined electrochemical and thermal effects [156,157]. Studies have shown that ohmic heating (220 V, 50 Hz) for periods over 3 min efficiently inactivates TI when compared to induction cooker or electric stove methods over 3 min. The residual trypsin inhibitory activity was 13% (ohmic heating), which is significantly lower compared to 19% using an induction cooker and an electric stove [157]. Additionally, lipoxygenase inactivation followed first-order kinetics during ohmic heating and conventional heating. However, a significant variation in rate constants was observed. Studies have shown that the electric field has an additional effect on lipoxygenase inactivation, with approximately 5 times lower D values. This means that for the same inactivation degree, the time required for thermal treatment is much lower when an ohmic heating process is applied, thus reducing negative thermal effects on the other food components [158]. To date, risks including “cold spots” caused by uneven resistivity remain, complicating the industrial application of this process [159]. The selection of electrode materials is a very important factor when considering the industrial application of ohmic heating. An electrode can be designed for specific ohmic heating conditions only, as the electro-chemical interactions of food and electrode may depend on the electric field strength as well as the frequency and type of wave. The composition of food material is also a challenge for food processors while considering ohmic heating because different types of plant-based drinks may contain different components with different properties, e.g., electrical conductivity. The ohmic heating behavior of the food material in such cases becomes complex, and may lead to the underheating of components with low electrical conductivity or vice versa [160].

### 6.10. Packaging and Shelf Life

Plant-based drinks are packaged as needed for storage and distribution after processing, usually in plastic bottles or carton systems [1]. Plant-based drinks can also be drum-dried or spray-dried to produce stable powders that can be reconstituted into the desired product [161,162]. However, liquid plant-based drinks must be stabilized before drying to obtain a stable product [163]. For example, a calcium-fortified soy drink (200 mg/100 g) was formulated by adding water (85–90 °C), full-fat soy flour (10%), sucrose (2.75%), and soy protein isolate (2.25%). Following homogenization, the blend was twice clarified and pasteurized at 65 °C/30 min before refrigeration. The samples of the soy drink (45 °C) were adjusted to a pH of 8 before adding calcium lactogluconate (1.55%) and potassium citrate (1.25%). For successful calcium fortification, it is recommended to maintain a calcium-to-protein ratio < 38 mg/g and to use an appropriate sequestering agent at a molar ratio of 0.8/mole calcium [164].

Plant-based drinks are rich in nutrients and are an ideal medium for microbial growth. Therefore, their quality will be adversely affected by the rapid growth of microorganisms. Heat treatment has been used to extend the shelf life of plant-based drinks while increasing the total solid output and improving the flavor. Overheating will adversely affect the development of nutrients (vitamins and amino acids), browning, and the development of cooked flavors [165]. In order to eliminate or reduce the destructive impact on plant-based drinks, various time and temperature combinations have been practiced to obtain the best quality products. Different heat treatments, such as pasteurization (heating to below 100 °C to destroy pathogenic microorganisms), container sterilization (121 °C for 15–20 min to achieve commercial sterility), and ultra-high-temperature treatment (at high temperatures of 135–150 °C for several seconds) have been widely studied [166]. Ultra-high-temperature treatment involves direct heating methods, including steam injection, or indirect heating in plate or tube heat exchangers. After any of the above treatments, packaging needs to be carried out under sterile conditions to maintain sterility. After pasteurization, plant-based drinks need to be stored under refrigerated conditions, while after sterilization in a container or ultra-high-temperature sterilization treatment, the plant-based drinks can be stored at room temperature for several weeks [7]. The effect of pasteurization on soybean drinks has been studied, and the results showed that pasteurized soybean drinks can be stored for 3 days after being heated at 60 °C for 30 min without obvious deterioration, and sterilized soybean drinks can be stored for 1 year after being heat treated at 120 °C for 5 min [167]. Heat treatment is widely applied to extend the shelf life of plant-based drinks, such as soybean drinks and peanut drinks. However, the existence of medium and high starch concentrations in oat drinks and rice drinks restricts their application [7]. Therefore, these types of plant-based drinks need to apply non-heat treatment technology to extend their shelf life. Some non-heat treatment technologies, including high-pressure throttling, ultra-high-pressure homogenization (UHPH), and high-pressure processing, have been studied to extend the shelf life of plant-based drinks [126,168,169,170]. The effect of ultra-high-pressure homogenization combined with heat treatment on the microbial stability of almond drinks has been studied. The microbiological analysis, physical stability, and chemical analysis results showed that when comparing UHPH treatment with pasteurization and UHT treatment, the product quality was higher compared to samples treated with pasteurization or UHT. After incubation at 30 °C for 20 days at a pressure of 300 MPa, a temperature of 65 and/or 75 °C, and a holding time of less than 0.7 s, the product showed no bacterial growth [171]. These results show the potential of non-heat treatment for extending the shelf life of plant-based drinks. However, the antiseptic effects and mechanisms of pulsed electric fields and other non-thermal technologies, such as pulsed light and ultrasound, on different types of plant-based drinks need to be further explored.

## 7. Challenges Facing Plant-Based Drink Processing

The plant-based drink processing industry must address challenges, including the beany smell and urease in soy drinks, which reduce their culinary appeal, and starch granules in oat drinks, which affect their smooth taste. The challenges faced during plant-based drink processing include product safety, nutrition, and stability.

### 7.1. Plant Cell Wall Tissue Limits the Dissolution of Endogenous Nutrients

The health benefits of foods depend on their individual components and their structure (or matrix). Food structure plays a regulatory role in digestion and subsequent physiological metabolic reactions and affects nutrient dissolution during processing. Plant-based food structure is determined by the presence of a cell wall, which is composed of a polymeric structure with a skeleton composed of cellulose as the core and a hydrated gel matrix composed of polysaccharides [172]. The intercellular layer is rich in pectin. As the outermost layer of a plant cell, it also connects juxtaposed plant cells together [172]. Plant cell wall structure and composition vary depending on plant species, tissue distribution, and growth stage [173]. Legumes and other dicot seed cell walls are rich in pectin and xylan, whereas cereal cell walls and those of other monocots are low in pectin but contain arabinoxylan and/or mixed junctions containing b-D-glucan [174]. The dissolution of proteins, oils, dietary fibers, and small-molecule phytochemicals in plant cells is greatly limited by cell walls. Therefore, to increase the solid content of plant-based drinks (by increasing nutrient dissolution), it is crucial to optimize and innovate technologies applied during processing stages, including heat treatment, extrusion, fermentation, grinding, and homogenization [175]. By changing the structure of plant-derived foods and using treatments such as heat and pressure, cell walls can be broken into porous structures, releasing endogenous nutrients from inside cells, thereby improving the dissolution rate of proteins, lipids, polyphenols, and flavonoids in plant-based drinks.

### 7.2. The Challenge of Efficiently Removing Consumer Health Risk Factors from Plant Sources

Heat-sensitive consumer health risk factors (including cyanogenic glycosides and antivitamin factors) can be eliminated via heat treatment [176]. Major allergens in plant-based drinks include soybean proteins 7S and 11S; peanut proteins 7S, 11S, and 2S; and gluten in grain seeds. Currently, the most widely used methods of elimination include enzymatic hydrolysis, fermentation, high static pressure, and irradiation [177]. In grain seeds, phytic acid is mainly concentrated in the outer shell and germ part and is relatively low in the endosperm. The phytic acid content in soybean seed coats is low, with only 1% located in the germ and 99% existing in the cotyledons. Therefore, depending on the raw material, phytic acid can be selectively reduced using different methods, including shelling, water blanching, and removal by soaking. Phytic acid can also be removed by cooking, germination, microbial fermentation, and the addition of exogenous phytase [178].

### 7.3. The Control of System Stability

As multiphase dispersion systems, plant-based drinks contain various colloidal substances, including proteins, lipids, oil bodies, polysaccharides, polyphenols, phytic acid, derivatives of these compounds, plant tissue fragments, and other particles. These systems are thermodynamically unstable and prone to phase separation [179]. The main factors underlying the physical instability of plant-based drinks include the following: (A) Force-induced separation: the dispersed particles in plant-based drinks have different densities than those in the water phase, causing them to move in response to gravitational forces. (B) Particulate matter less dense than water, such as grease bodies or fat droplets, tends to float, whereas denser particulate matter, such as plant cell debris, starch granules, protein aggregates, and calcium carbonate granules, tends to sink. (C) Aggregation in plant-based drinks: electrostatic and hydrophobic interactions occur between lipids, protein-embedded fat droplets, protein particles, and/or plant cell debris. When the interaction force between colloids changes, aggregation occurs easily, mainly through flocculation and coalescence. Plant-based drinks are, thus, prone to stratification and precipitation during storage, which can be detrimental to taste. Plant-based drinks can also experience system instability owing to various chemical or biochemical processes, including oxidation, hydrolysis, and microbial action, which reduce the stability and safety of plant-based drinks and produce an unpleasant volatile odor [3,180].

### 7.4. Adjustment of System Flavor

Plant-based drinks, which are commercially popular, are generally roasted, giving them nutty, burnt, and sweet aromas. Owing to the unique properties of their raw materials, different plant-based drinks, including coconut, bean, flax, and grain, also have unique flavors and aromas. Plant-based drinks may also contain unappealing flavor attributes. In addition to rancid flavors caused via the oxidation of polyunsaturated fatty acids, they may also have unique grass, raw, and earthy flavors derived from plant raw materials and mainly attributable to low-molecular-weight alcohols, aldehydes, ketones, and furans. Typical off-flavor substances include 1-octen-3-ol (mushroom flavor), hexanal (grass flavor), (E)-2-octenal (cucumber flavor), and (E,E)-2,4 -decadienal (oily), and 2-pentylfuran (grass flavor) [181]. Some substances do not individually produce obvious odors, but their odorant capacities are enhanced in mixtures. Therefore, the material basis of odors is also difficult to define in studies of plant-based drinks. Regarding the formation mechanism of off-flavor components, using soy drink as an example, it is generally believed that C6 aldehyde is an important component of the flavor of legume foods and is derived from the enzymatic oxidation pathway catalyzed via lipoxygenase, with linoleic acid and linolenic acid being the main precursors [182]. Linoleic acid is oxidized via lipoxygenase to produce 9- or 13-hydroxy-linoleic acid hydroperoxide, which further reacts with hydroperoxide lyase to form hexanal, (E)-octenal, and (E,E)-2,4-Decadienal, etc. When linolenic acid is used as a substrate, (E)-2-hexenal and (E,Z)-3,6-nonadienal are formed [183].

Hexanal and (E)-2-hexenal are the main grassy-flavor-contributing components produced via the lipoxygenase pathway [184]. Non-enzymatic reactions also occur in soybean pulping. Lipids, proteins, carbohydrates, and other precursor substances can form different oxidation intermediates, including hydroperoxides and free radicals, ultimately leading to the production of odors. These all require the regulation of systemic flavor in plant-based mill production processes [185].

### 7.5. Control of Spoilage

Because plant-based drinks are rich in carbon and nitrogen sources, microorganisms can easily reproduce and cause spoilage. The main reasons include the following: (A) Mildewed or spoiled plant-based drink raw materials make it difficult to meet the microbial indicator specifications of the final product. (B) Improperly selected plant-based drink sterilization methods. Pasteurization can kill pathogenic bacteria and most non-pathogenic bacteria in plant-based drinks, but these products retain small proportions of heat-resistant bacteria and spores, resulting in shorter shelf lives. Ultra-high-temperature instantaneous sterilization methods coupled with aseptic canning, canning first, and then high-temperature and high-pressure sterilization are commonly used and are safer sterilization methods for plant-based drink processing [186]. (C) Problems arise during sterilization, and improper process control, such as excessive product accumulation during high-temperature and high-pressure sterilization, incomplete sterilization, ultra-high-temperature instantaneous sterilization coupled with aseptic canning process, and improper cleaning of sterilizers and pipelines, may cause corrosion and deterioration of final products during storage [187]. Therefore, it is necessary to develop green and efficient methods and technologies to control the production of harmful microorganisms in plant-based drinks.

## 8. Conclusions

Plant-based drinks represent an emerging healthy food with a high nutritional value, which exert a positive impact on human health through long-term consumption. The flavor quality of plant-based drinks is an important evaluation standard for consumers, but most plant-based drinks are difficult for consumers to accept because of their bitter and astringent flavors. Improving the flavor of plant-based drinks is an important bottleneck that the industry needs to overcome. Therefore, the scientific issues in using food flavoromics research methods to conduct in-depth research into plant-based drink flavors should focus on new extraction technologies for plant-based drink flavors, the analysis of substances contributing to aromas, pleasant aroma and off-odor formation mechanisms, aroma optimization, and odor control technologies. Additionally, raw materials containing low levels of odor components should be screened, and novel processing technologies should be explored in depth, combined with the improvement and optimization of odor removal or burial processes, to create new plant-based drink options rich in nutrition and good flavor, expand plant-based drink marketing opportunities, and provide necessary theoretical and technical support for development within the plant-based drink processing industry.

In the burgeoning landscape of plant-based drinks, several impending challenges merit careful consideration for the sustainable evolution of this dairy alternative. Environmental sustainability remains a paramount concern, demanding the judicious management of resource-intensive crop production. Supply chain resilience and nutritional adequacy require meticulous attention to mitigate vulnerabilities associated with disruptions and nutritional disparities. Furthermore, allergen labeling precision, product quality enhancement, and adherence to evolving regulatory frameworks necessitate ongoing vigilance. Competition-driven market dynamics, coupled with consumer education imperatives, underscore the need for strategic differentiation and consumer enlightenment. Infrastructure development, waste management solutions, and the recognition of regional variations in preferences further compound the multifaceted nature of these challenges. Addressing these complexities while upholding ethical and labor standards constitutes an imperative dimension of sustainable plant-based drink development, necessitating a collaborative effort among stakeholders to navigate these intricacies effectively.

## Figures and Tables

**Table 1:** Plant-based drink classification by raw material source.

Classification	Raw Material Sources	References
Nut-based plant drinks	Almonds, pistachios, walnuts, cashews, etc.	[7,15]
Soy-based plant drinks	Peas, chickpeas, soybeans, peanuts, cowpeas, etc.	[7]
Seed-based plant drinks	Sesame, flax, hemp, pumpkin seeds, etc.	[7,15]
Cereal-based plant drinks	Millet, corn, barley, sorghum, wheat, etc.	[7,15]
Pseudo-cereal-based plant drinks	Quinoa, moss bran, etc.	[7]

**Table 2:** General composition of different plant-based drinks.

Drink Type	Protein (g)	Total Fat (g)	Ash (g)	Total Carbohydrates (g)	Fiber (g)	References
Soy	2.78	1.96	0.75	3	<0.75	[20,21]
Coconut	2.02	21.3	0.97	2.81	-	[20,22]
Oat	0.8	2.75	0.79	5.1	<0.75	[20,23]
Flaxseed	-	1.04	-	0.42	-	[20,24]
Rice	0.42	1.04	0.34	9.58	-	[20,25]
Cashew	2.2	5.29	-	5.73	0.4	[20,26]
Almond	0.66	1.56	0.6	0.67	<0.75	[20,26]

**Table 3:** Main fatty acid components of plant-based drinks.

Drink Type	Main Fatty Acid Components %							Reference
	Saturated Fatty Acid				Unsaturated Fatty Acid			
	C12:0	C14:0	C16:0	C18:0	C18:1n − 9	C18:2n − 6	C18:3n − 3	
Soy	-	-	9.8	3.7	21.9	53.7	9.9	[37]
Coconut	50.0	17.3	7.5	2.7	0.01	0.77	-	[38]
Oat	-	-	5.09	1.81	45.1	23.7	0.234	[39]
Walnut	-	-	8.0	3.0	18.0	59.0	6.0	[40]
Peanut	-	-	16.63	4.88	42.63	15.56	-	[41]
Flaxseed	-	4.59	20.4	6.65	27.59	24.24	7.01	[42]
Almond	-	-	14.6	10.8	54.0	15.4	-	[39]

**Table 4:** Main mineral components of plant-based drinks.

Drink Type	Calcium (mg)	Iron (mg)	Phosphorus (mg)	Magnesium (mg)	Potassium (mg)	Sodium (mg)	Zinc (mg)	Reference
Soy	155	0.37	46	17.5	118	39	0.26	[20,21]
Coconut	18	3.3	96	46	220	13	0.56	[20,22]
Oat	148	0.26	89	5.9	148	42	0.09	[20,23]
Flaxseed	125	0.15	62	-	-	33	-	[20,24]
Rice	125	0.3	62	-	-	42	-	[20,25]
Cashew	9	1.51	66	35	84	51	1.26	[20,26]
Almond	158	0.12	19	8.2	49	59	0.08	[20,26]

**Table 5:** Main vitamin components of plant-based drinks.

Drink Type	Thiamin (mg)	Riboflavin (mg)	Vitamin B-6 (mg)	Folate (µg)	Vitamin B-12 (µg)	Retinol (µg)	Vitamin D (µg)	Reference
Soy	0.044	0.331	0.036	16	1.33	89	4.63	[20,21]
Coconut	0.022	-	0.028	14	-	-	-	[20,22]
Oat	0.04	0.281	0.006	<6	0.51	85	1.7	[20,23]
Flaxseed	-	-	-	-	0.62	-	1.05	[20,24]
Rice	-	-	-	-	0.62	-	1.05	[20,25]
Cashew	-	0.015	0.053	-	-	-	-	[20,26]
Almond	0.005	0.083	<0.01	<6	0.45	61’	1.59	[20,26]

**Table 6:** Total phenolic compounds and phytosterol compositions of plant-based drinks.

Drink Type	Total Phenolic Compounds	β-Sitosterol (mg/100 mL)	β-Sitosterol-β-D-Glucoside (mg/100 mL)	Campesterol (µg/100 mL)	Stigmasterol (µg/100 mL)	Reference
Almond	1.24 mg GAE/L	2.5 ± 0.1	13 ± 2	62 ± 4	1915 ± 109	[51,52,53]
Hazelnut	130.42 mg GAE/100 mL	-	-	-	-	[54,55]
Sesame	4 mg GAE/g	-	-	-	-	[56,57]
Soy	8.79 mg GAE/100 g	2.5 ± 0.5	4.9 ± 2.1	1290 ± 291	998 ± 111	[51,58,59]
Rice	122.05 mg GAE/100 mL	0.51 ± 0.07	2.4 ± 0.6	260 ± 28	234 ± 23	[51,60]
Cashew	-	2.7 ± 0.4	>60	279 ± 44	15 ± 1	[51]
Oat	15 mg GAE/100 mL	2.1 ± 0.2	26 ± 4	475 ± 30	182 ± 16	[51,61]

## Data Availability

Data is contained within the article.
